# Clinical profile of orofacial infections: An experience from two primary care dental practices

**DOI:** 10.4317/medoral.17664

**Published:** 2012-02-09

**Authors:** Marina G. Kudiyirickal, Frank Hollinshead

**Affiliations:** 1 BDS, PhD Student, Department of Dentistry, Charles University in Prague, Czech Republic; 2 BDS, LDSRCS, Alchemy Dental Practice, Staffordshire, West Midlands, UK

## Abstract

Objectives: Orofacial infections are common reasons for dental consultations worldwide. However, there is scarcity of data on clinico-epidemiological profiles reported from primary care dental practices. To address this issue, a study was done to characterize the clinical pattern, age groups affected and sex predilection of orofacial infections in the primary care dental practice. Study design: Clinical data was evaluated from random electronic files of patients for whom antimicrobials were prescribed at two Dental Practices in UK between January 2009 and December 2010. Results: 200 case records were studied. 104 (52%) cases were females. Mean age was 37.2 (+/-15.1) years. 107 (53.5%) cases belonged to age group 21-40 years. Posterior teeth were involved in 112 (56%) cases. Types of disease were as follows: dentoalveolar abscess 63(31.5%), pulpitis 27(13.5%), apical periodontitis 21(10.5%), pericoronitis 21(10.5%), dry socket 13(6.5%), periodontitis 9(4.5%) infected root stump 5(2.5%), facial swelling 5(2.5%) and infections unspecified 36(18%) cases. Conclusions: Orofacial infections affect both sexes equally. 21-40 years is the commonest age-group affected. Dentoalveolar abscess is the commonest infection followed by unspecified infections and pulpitis.

** Key words:**Orofacial infections, primary care dental practice, dentoalveolar abscess and pulpitis.

## Introduction

Oral and maxillofacial infections are common health problems and frequent causes of dental consultation worldwide. More than 500 bacterial species are known to constitute normal oral microbiota (1). Microorganisms in the oral cavity have been implicated as causative agents in dental caries, pulpitis, abscess, periodontal disease and halitosis, bacterial endocarditis, aspiration pneumonia, osteomyelitis in children, preterm low birth weight, coronary heart disease and cerebral infarction (2).

Orofacial infections may be odontogenic or non odontogenic in nature and the vast proportion of odontogenic infections are caused by the endogenous bacterial microbiota in the oral cavity (3) while non odontogenic infections vary depending on the nature and site of infection (4). Unlike odontogenic infections the non odontogenic infections do not affect the teeth. Mucosal infections by bacteria account for the majority of the oral non odontogenic infections (5).

Orofacial infections may lead on to significant complications unless they are managed timely and appropriately. Morbidity and mortality related to these infections depend on the site of involvement and the degree of spread to other tissues. A majority of these infections are 

confined to the dentoalveolar and/ or facial tissues at the time of presentation, but may spread to local, regional or distal sites if treatment is delayed (6).

In order to manage these infections scientifically, correct identification of the aetiology and pathology of the disease is important. Appropriate choice and duration of antimicrobial prescription for these infections rely upon the age, systemic infection and other co-morbidities of the patient. Analysis of etiopathogenesis and presentations of orofacial infections, in a general dental practice would help to understand the clinical spectrum of these important illnesses and to tailor the right treatment strategy for patients in future. With this purpose, a retrospective study was conducted to determine the types of disease, age groups affected, sites involved and gender predilection among patients treated for orofacial infections in two primary care dental practices.

## Material and Methods

A retrospective study of patients who were treated with antimicrobials in two dental practices in West Midlands, UK between January 2009 and December 2010 was done. From the clinical database of patients treated during this period, every third patient who was prescribed an antimicrobial was identified from prescription records. The clinical data of these patients were retrieved from computer records. Demographic and clinical parameters like age, gender, smoking status, alcohol consumption, relevant medical history, teeth affected, clinical diagnosis and type of treatment provided were identified and recorded.

Cases with incomplete records where it was difficult to determine basic data like clinical diagnosis and type of antimicrobial prescribed were excluded from the study. The data retrieved were entered into an excel sheet for the purpose of analysis. The study protocol was formally approved by the institutional review board.

Statistical analysis was performed using computer software SPSS for Windows, version 11.5 (SPSS Inc, Chicago, IL, USA). Categorical variables were compared with chi square test. Continuous variables were compared by unpaired ‘t’ test and are presented as mean (+/- SD). Level of statistical significance was set at P <0.05.

## Results

226 case records were retrieved from computer database where antimicrobials were prescribed. After excluding the data of incomplete case records (that did not provide sufficient data for analysis), 200 cases were identified for final analysis. 104 (52%) were females and 96 (48%) were males. 56 (28%) were smokers, while 90 (45%) were non-smokers. No record of smoking status was available for 54 (27%) cases. 84 (42%) of the patients had history of alcohol consumption, while 60 (30%) had been non-alcoholics. 56 (28%) cases had no data on alcohol consumption.

Mean age of the study cohort was 37.2 (+/- 15.1) years. 65 cases (32.5%) belonged to the age group 21 – 30 years. The distribution of cases according to the age groups is shown in  Table 1.

**Table 1:**
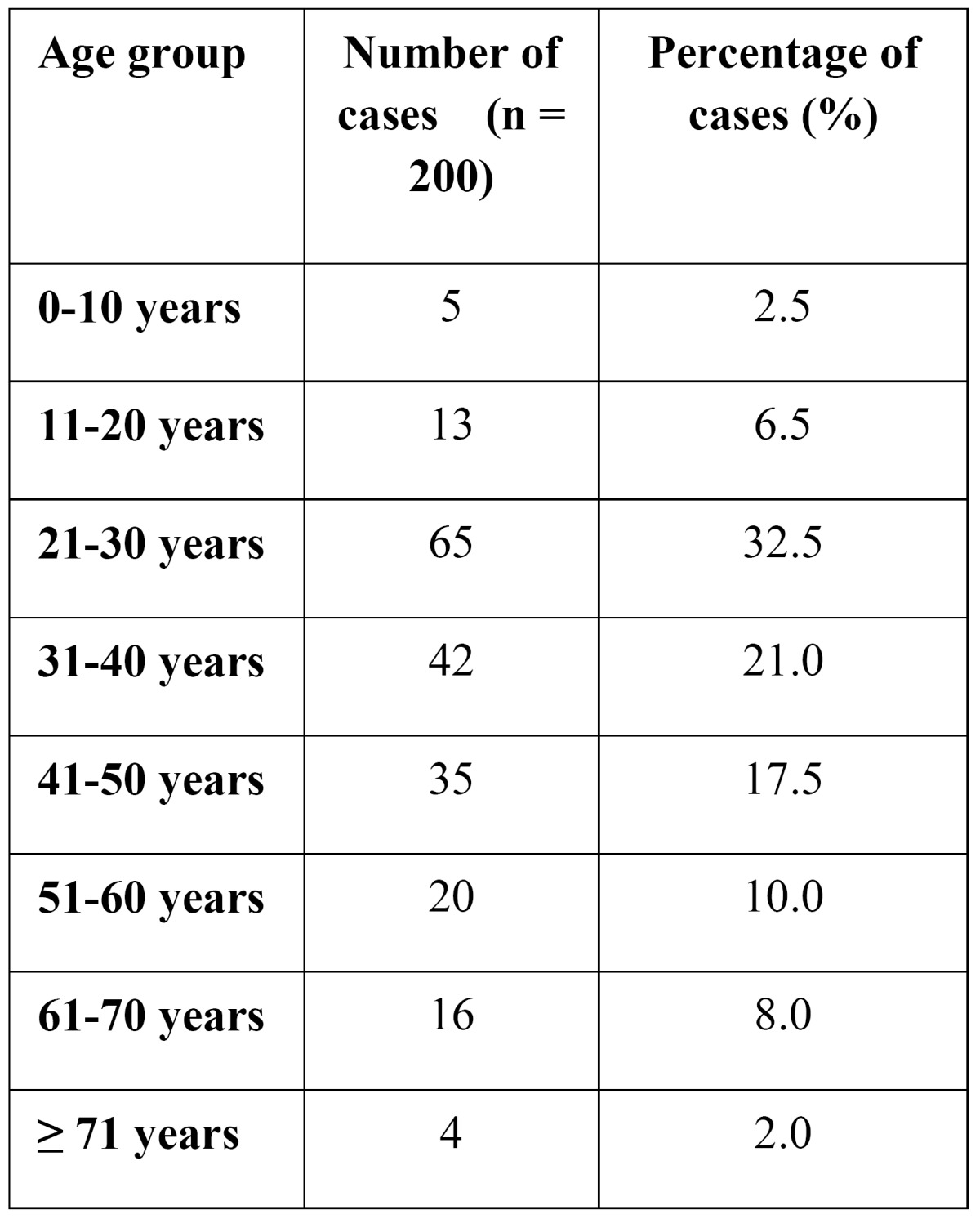
Age distribution of cases.

Teeth and site affected: Posterior teeth were most commonly infected accounting for a total of 112 (56%) cases, while anterior teeth and deciduous teeth constituted 10 (5%) and 5 (2.5%) cases respectively. Symptomatic involvement of multiple teeth was recorded in 28 (14%) cases. Lower right first molar tooth was the commonest offending tooth (8% of cases) followed by lower left third molar (7%) and second molar (6%). However, appropriate details were unavailable to identify the tooth and the site involved in 45 (22.5%) cases.

A large proportion of the infections where found to be in the lower posterior teeth, both right and left sides, with equal predilection 36 (18%) cases each, followed by upper posterior teeth with a predilection for left side (11.5%), upper right posteriors (8.5%), upper left anteriors (3%), deciduous teeth (2.5%), upper right anterior (1.5%) and the least was for lower right anterior teeth (0.5%).

Types of disease: Most common infection identified was dental abscess (31.5%), followed by pulpitis (13.5%), apical periodontitis (10.5%), pericoronitis (10.5%), dry socket (6.5%), periodontitis (4.5%), infected root stump (2.5%) and facial swelling (2.5%). However, in 36 (18%) cases the type of disease was not grouped under any of the above category (conditions such as acute necrotising ulcerative gingivitis (ANUG), gingival abscess, referred pain in the teeth secondary to maxillary sinusitis, anticipated infections in traumatic dental extractions or infective endocarditis prophylaxis). A gender specific distribution of the types of disease is shown in the  Table 2.

**Table 2:**
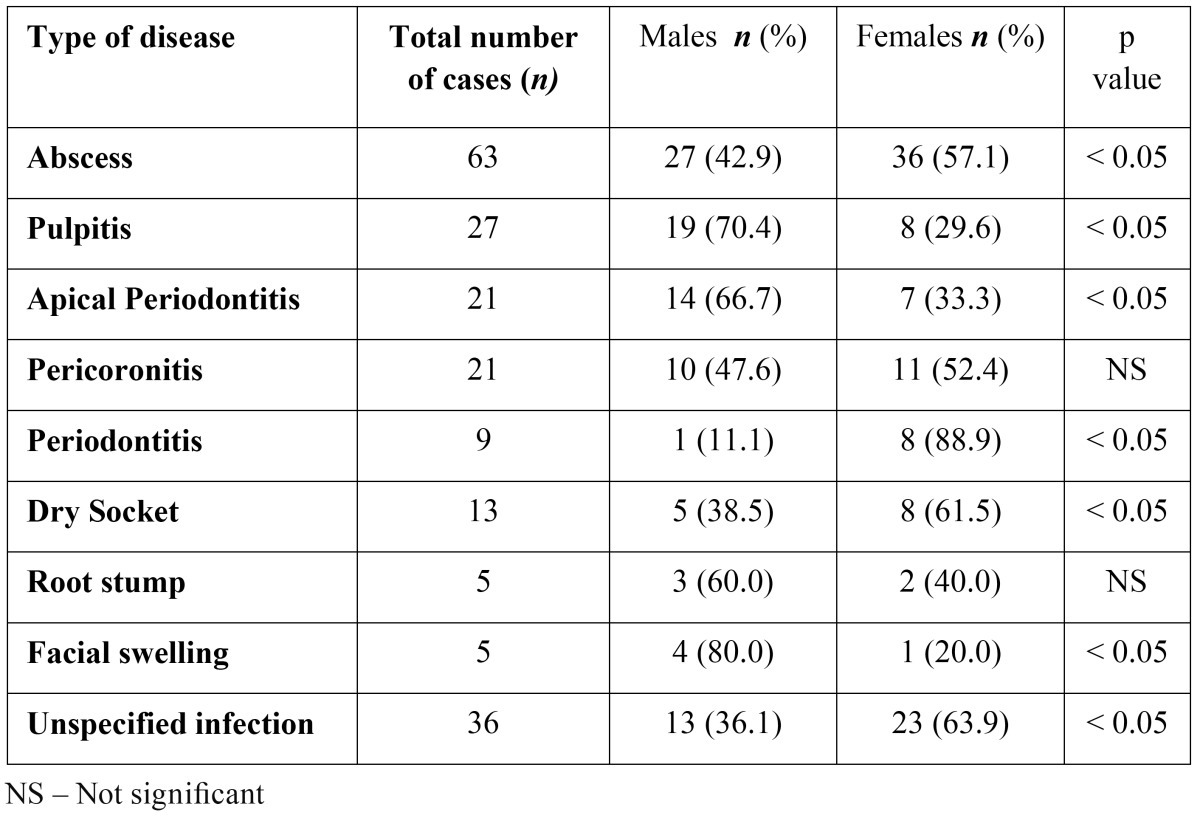
Gender specific distribution of types of disease.

An age specific distribution of disease types is shown in  Table 3.

**Table 3:**
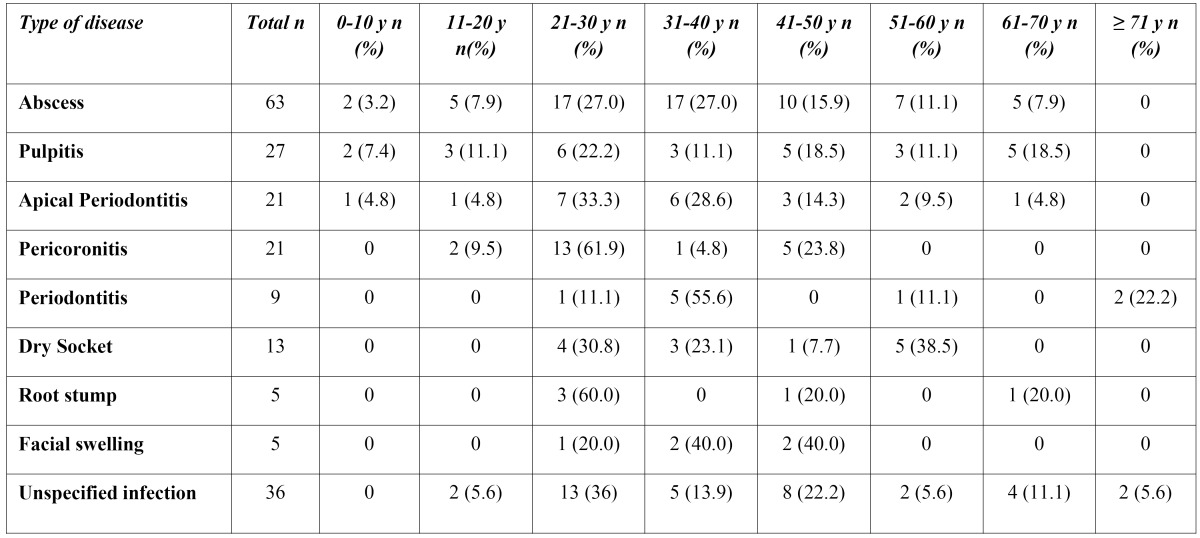
Age specific distribution of various disease types.

Periodontitis was found to be much more common among patients aged between 31 and 40 years.

Treatment modalities given to cases: 104 (52%) cases were given only antimicrobial treatment for their illnesses. Of the remaining cases, 17 (8.5%) were treated with dental extraction, 16 (8%) with wisdom tooth management, 14 (7%) with root canal treatment and 9 (4.5%) with alvogyl for dry socket, in addition to antimicrobial prescription. 21 (11.5%) cases received combination treatment with more than one of the above therapeutic measures along with antimicrobial prescription. 9 (4.5%) cases were referred for specialist opinion and management. In 8 (4%) cases the exact treatment modality, other than antimicrobial prescription, was not recorded in the database.

## Discussion

This study provides an overall picture of the types of oral and maxillofacial disease where antimicrobials were used in two primary dental practices. The gender and age predilection, and the common sites involved are also revealed.

There was no significant gender difference for clinic visit rate due to orofacial infections in this study cohort. This finding is similar to the earlier observation reported by YM Dailey et al (7). However, Akinbami et al. recently reported a female preponderance in their study (6). Geographical differences in the study cohorts may explain this disparity.

45% of cases in the study cohort were non-smokers. Smoking can adversely affect the oral bacterial flora and may predispose orofacial and periodontal disease (8). Because 28% of the cases did not have data on smoking status, we could not analyse if there was any difference in the pattern of disease in relation to smoking in the cohort. Alcohol consumption also predisposes to periodontal disease (9). Although 42% of cases reported alcohol consumption, 28% missed the data and hence we could not analyse the association between alcohol and disease pattern in this study.

Aging is associated with reduction in adaptive immunity and the elderly are more susceptible to oral and dental infections (10). However, the mean age of cases in our study was 37.2 (+/- 15.1 years) only. It is noteworthy that 107 (53.5%) cases belonged to the age group 20 to 40 years in the study cohort. Heightened oral health awareness among younger population may be one reason for this observation. Future studies may identify the exact cause of this interesting observation.

Dentoalveolar abscess is reported to account for 25% of emergency dental visits for odontogenic infections (11). In this cohort, dental abscess was the commonest lesion for which antimicrobials was prescribed (31.5% of cases). 34.3% of antimicrobial prescriptions for emergency dental visits were for dentoalveolar abscess in the large British study reported by YM Dailey et al (7). Significantly higher numbers of females had dentoalveolar abscess in the present study. Similar observation was made previously by Akinbami et al (6), although a male preponderance was reported by Rega AJ et al (12).

39.2% of antimicrobial prescriptions were for pulpitis in the series reported by YM Dailey et al (7). Only 13.5% of cases in the present study had pulpitis as the diagnosis. Rational management of pulpitis does not necessitate antimicrobial prescription and the heightened awareness of this fact recently among the dental professionals might have attributed to the decrease in antimicrobial usage for the disease explaining this discrepancy.

Significantly higher proportion of males (compared to females) had apical periodontitis in the present study although a female preponderance of apical periodontitis was reported in the study by Akinbami et al (6). The proportion of periodontitis cases was much less in this cohort compared to the cohort studied by Akinbami et al. Low prevalence of periodontal disease was also reported by YM Dailey et al (7). The prevalence of pericoronitis in the present study was comparable to that observed by Akinbami et al. However, there was no statistically significant difference in the sex ratio as observed by Akinbami et al (6).

Posterior teeth were most commonly involved with infection in this study. This observation is similar to those by other investiga-tors (6,13-15). Posterior teeth have wider surface area and they are utilized for mastication and are subjected to more occlusal stress, micro/ macrotrauma, caries, impaction and stagnation of food debris and have reduced accessibility to thorough hygiene. Treatment failure/ recurrent treatment are more common with posterior teeth. All these factors make them more vulnerable to infections.

A majority of cases in the present study received only antimicrobial prescription as the sole treatment modality. This observation is similar to that reported by YM Dailey et al (7). Dental extraction rates for treatment also was comparable. However, Akinbami et al. reported that 92% of cases in their study cohort were treated by dental extraction (6). Marked difference in the disease pattern among the patients from different studies and the differences in clinical practice in different countries may be the reason for this discrepancy.

In conclusion, dentoalveolar abscess was the commonest infection for which antimicrobials were prescribed. Antimicrobial prescription rates for management of pulpitis by dental professionals are less these days. People in their third and fourth decades of life are the commonest age groups affected by orofacial infections and there is no sex predilection for the disease.
